# The Role of Iron in Neuronal Homeostasis: A Double-Edged Sword

**DOI:** 10.3390/cells15110999

**Published:** 2026-05-29

**Authors:** Bibiana Sgalletta, Francesco Agostini, Marco Bisaglia

**Affiliations:** Department of Biology, University of Padua, Via Ugo Bassi 58/B, 35131 Padua, Italy; bibiana.sgalletta@phd.unipd.it (B.S.); francesco.agostini.2@unipd.it (F.A.)

**Keywords:** iron, neuroinflammation, neurogenesis, neurodevelopment, neurodegenerative disorders

## Abstract

Iron is an essential micronutrient that plays a central role in numerous biological processes. Despite its relatively low abundance in the human body, iron is particularly critical for brain function. Systemic and cerebral iron homeostasis is tightly regulated through coordinated mechanisms involving absorption, transport, storage, and recycling. Within the brain, iron metabolism is further controlled by the blood–brain barrier and specialized neural cell populations, including neurons, astrocytes, oligodendrocytes, and microglia. Iron is indispensable for neurodevelopment, supporting neurogenesis, myelination, and neurotransmitter synthesis. However, both iron deficiency and iron overload have detrimental consequences. Early-life iron deficiency disrupts neural development and leads to long-lasting cognitive, motor, and behavioral impairments, whereas excessive iron accumulation promotes oxidative stress, ferroptosis, and neuroinflammation. These mechanisms have been described to contribute to the pathogenesis of major neurodegenerative disorders, including Alzheimer’s disease, Parkinson’s disease, neurodegeneration with brain iron accumulation, and amyotrophic lateral sclerosis. This review first outlines systemic and brain iron metabolism, highlighting how neural cells regulate homeostasis. Next, it examines iron’s physiological roles, particularly in neurogenesis and neurodevelopment. Finally, it explores iron’s involvement in neurodegenerative diseases, emphasizing neuroinflammation as a primary mechanism of iron toxicity.

## 1. Introduction

While iron is the most abundant element on Earth, it represents a negligible fraction of human biology, totaling approximately 4 g of body mass. This iron is distributed primarily within erythrocytes (60–70%) and storage sites such as macrophages, bone marrow, and the liver (20–30%). The remainder is found in myoglobin (5–10%) and enzymes (1%) [1]. The daily requirement for this element is estimated at 25–30 mg, the majority of which is used to synthesize hemoglobin for new erythrocytes [2]. Despite its relatively low concentration in the body, iron is indispensable for fundamental cellular processes across all tissues and plays a particularly critical role in neuronal function. Iron deficiency is among the most prevalent micronutrient deficiencies worldwide and has been associated with neurodevelopmental impairments, including deficits in psychomotor performance and cognitive function [3,4]. Conversely, excessive iron is also harmful, as iron overload can promote oxidative stress, mitochondrial dysfunction, and ultimately cellular injury or death, including neuronal loss. Consistent with this dual role, abnormal iron accumulation has been implicated in the pathogenesis of several neurodegenerative diseases, such as Alzheimer’s disease, Parkinson’s disease, amyotrophic lateral sclerosis, and Huntington’s disease [5].

In this review, we will first describe the processes involved in systemic and brain iron metabolism by focusing on the participation of different neural cells in homeostasis regulation. We will then describe the physiological functions of iron with particular emphasis on neurogenesis and neurodevelopment. In the last part of this review, the role of iron in the pathogenesis of different neurodegenerative disorders will be considered, focusing on neuroinflammation as a common mechanism for its toxicity.

## 2. Systemic Iron Homeostasis

The strategic regulation of iron at a systemic level is critical for organismal health. Iron is predominantly recycled from senescent erythrocytes by macrophages, with a smaller contribution arising from intestinal absorption [6,7]. Notably, a substantial fraction of total body iron (~600 mg) is sequestered within tissue macrophages [2], which dynamically adjust iron release in response to systemic demands through interactions with hepcidin and ferroportin (FPN1), as discussed below. Splenic and hepatic macrophages phagocytose and degrade aged or damaged erythrocytes, thereby recovering iron for reuse, primarily to support hemoglobin synthesis during erythropoiesis [2].

Since mammals have no effective physiological mechanism for iron excretion [8], its absorption from the diet, subsequent transport, secure storage, and efficient recycling must be finely controlled. These pathways have evolved to precisely meet the body’s metabolic needs while preventing the accumulation of free iron that could lead to widespread cellular damage.

### 2.1. Iron Absorption, Transport, and Storage

**Figure 1:**
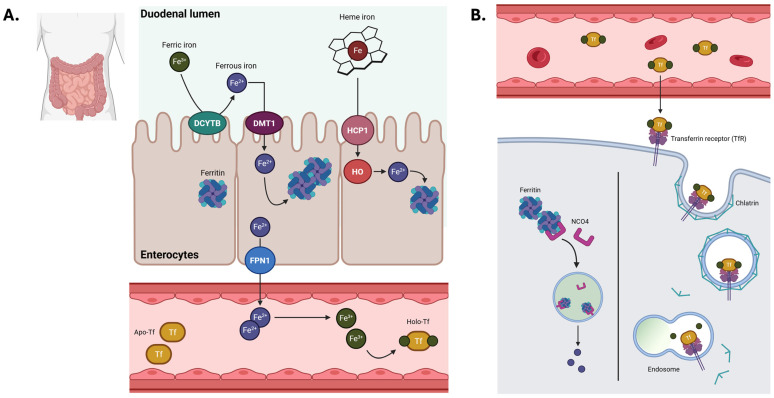
Mechanisms of dietary iron absorption and cellular metabolism (**A**) At the level of the duodenal enterocytes, dietary non-heme ferric iron (Fe^3+^) is reduced to ferrous iron (Fe^2+^) by duodenal cytochrome b (DCYTB), a ferrireductase located on the apical membrane of enterocytes. Ferrous iron is then transported into the cell via divalent metal transporter 1 (DMT1), which specifically mediates the uptake of divalent metal ions across the apical membrane. Heme iron, derived primarily from animal sources, is internalized at the apical surface of enterocytes via heme carrier protein 1 (HCP1). Inside the cell, heme is degraded by heme oxygenase (HO), releasing ferrous iron (Fe^2+^). The released iron subsequently enters the intracellular labile iron pool, where it becomes available for metabolic processes. Once inside the enterocyte, iron can either be stored in ferritin or exported across the basolateral membrane by ferroportin (FPN1). During export, ferrous iron is oxidized back to ferric iron (Fe^3+^) by the ferroxidases hephaestin and ceruloplasmin, a necessary step for its binding to transferrin (Tf). In the bloodstream, two molecules of ferric iron (Fe^3+^) bind to the transport protein Tf, which delivers iron to peripheral tissues. Tf-bound iron circulates in a soluble and non-toxic form, limiting the formation of harmful reactive oxygen species. (**B**) Ferritin serves as the major intracellular iron storage protein and sequesters excess iron in a non-toxic, bioavailable form. Ferritin is degraded inside cells via nuclear receptor coactivator 4 (NCOA4)-mediated ferritinophagy, resulting in the release of ferrous iron. This process allows stored iron to be mobilized when cellular iron demand increases. Iron is taken up by cells through the Tf receptor (TfR), which binds to holo-transferrin (iron-loaded Tf) and internalizes the complex via a clathrin-mediated endocytosis process. Within the acidic environment of the endosome, Fe^3+^ dissociates from Tf and is reduced to Fe^2+^. The released ferrous iron is then transported into the cytoplasm via DMT1. Tf and TfR1 are subsequently recycled back to the plasma membrane, where Tf is released into the circulation for reuse.

As outlined in Figure 1A, the absorption of dietary iron occurs primarily at the apical surface of mature enterocytes in the duodenum [9]. Dietary iron exists in two forms: non-heme (ionic) and heme iron. Non-heme iron, typically in its oxidized ferric (Fe^3+^) state, must first be reduced to its ferrous (Fe^2+^) form by the enzyme duodenal cytochrome b [9]. This more soluble ferrous iron is then transported into the enterocyte by the divalent metal transporter 1 (DMT1) [9]. Heme iron is absorbed into the enterocyte via heme carrier protein 1, where it is then catabolized by heme oxygenase to release ferrous iron, which subsequently joins the common intracellular iron pool [9]. Once inside the cell, iron can either be stored in ferritin or exported across the basolateral membrane into the bloodstream via the iron exporter FPN1 [9].

Upon entering circulation, iron is immediately oxidized back to its ferric (Fe^3+^) form by ceruloplasmin and binds to the transport protein transferrin (Tf) [8]. Interestingly, through its interaction with ceruloplasmin, copper takes part in iron homeostasis. In fact, ceruloplasmin exhibits a copper-dependent oxidase activity, which transforms Fe(II) into Fe(III), assisting in such a way the Tf-mediated transport of iron in the plasma [10]. Each Tf molecule can carry two ferric ions, which are then delivered to various tissues. There, the binding of iron-laden transferrin (holo-Tf) to the Tf receptor (TfR) promotes the clathrin-mediated endocytosis (Figure 1B), leading to the internalization of the entire complex into an endosome [8]. Inside the acidic environment of the endosome, iron dissociates from Tf, is reduced to Fe^2+^, and transported into the cytosol by DMT1 [8].

To prevent toxicity from free iron, cells safely sequester excess iron in a redox-inactive form within ferritin, the primary intracellular iron storage protein [11]. Ferritin is a spherical complex composed of 24 subunits of ferritin heavy chain (FTH) and ferritin light chain (FTL) that can store up to 4500 iron atoms in a non-toxic ferric form [11]. The FTH/FTL ratio of ferritin is tissue-specific [11]. Organs with high iron-storing capacity, such as the liver and spleen, are predominantly composed of FTL (up to 90%) due to its superior structural stability and iron-sequestering capacity. Conversely, tissues characterized by high iron-oxidation activity, such as the heart and brain, primarily utilize FTH, which acts as an antioxidant via its ferroxidase activity [11]. When the cytosolic level of iron is low, intracellular iron can be mobilized via ferritin degradation by nuclear receptor coactivator 4 (NCOA4) in a process termed “ferritinophagy” (i.e., lysosomal degradation of ferritin) [11]. A mitochondrial-specific ferritin (MtFt) is also present in specific tissues with high metabolic activity, such as the brain, testis, and heart. In these tissues, MtFt most likely plays a role in protecting mitochondria from iron-induced oxidative damage [12]. MtFt is structurally and functionally similar to FTH, although with slower ferroxidase activity [12].

Iron metabolism is coordinated by two regulatory systems. The first operates at the systemic level and relies on the activity of hepcidin, which participates in regulating FPN1 levels. The second participates in iron homeostasis at the cellular level through post-transcriptional modulation of iron-regulatory proteins.

### 2.2. The Hepcidin-Ferroportin Regulatory Axis

**Figure 2:**
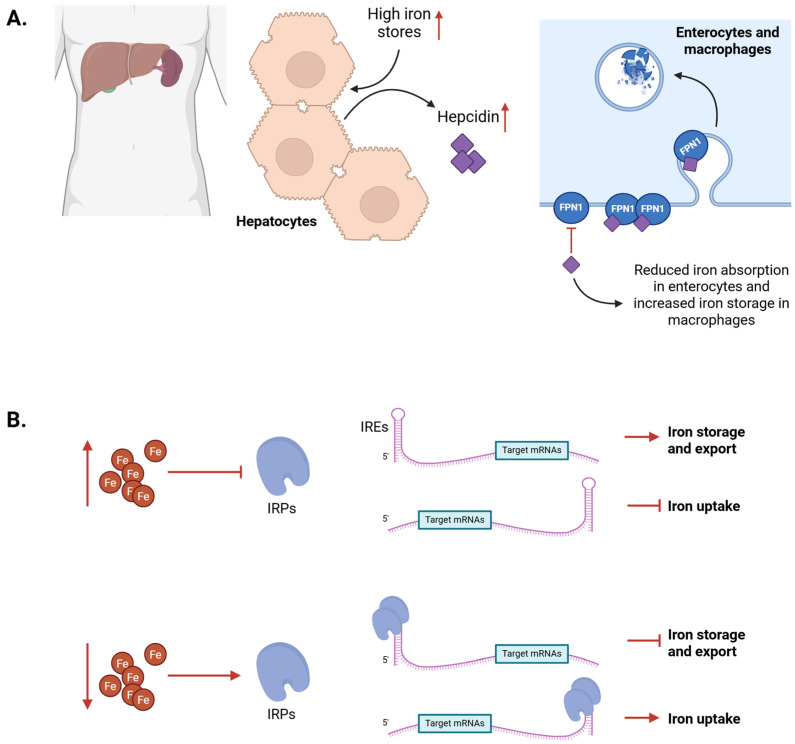
Feedback regulation of iron homeostasis (**A**) Hepatocytes produce hepcidin in response to systemic iron levels and inflammation. When body iron stores increase, hepcidin production also increases. Hepcidin acts on cells expressing ferroportin (FPN1), particularly enterocytes and macrophages and hepatocytes. After binding to FPN1, hepcidin induces its internalization and lysosomal degradation. Therefore, iron export into the bloodstream decreases, leading to reduced intestinal iron absorption and retention of iron within macrophages and hepatocytes. Conversely, when systemic iron levels are low or erythropoietic demand is increased, hepcidin synthesis decreases, allowing FPN1 to remain on the cell surface and promoting iron release into the circulation. (**B**) The IRP/IRE system is a major post-transcriptional regulatory mechanism controlling intracellular iron metabolism. Iron regulatory proteins (IRPs) are active when intracellular iron levels are low. Under low iron conditions, IRPs bind to iron-responsive elements (IREs) located in the untranslated regions (UTRs) of specific mRNAs involved in iron uptake, storage, and utilization. When intracellular iron levels are high, IRPs do not bind IREs. The regulatory effect depends on the position of the IRE within the mRNA: when IREs are located in the 5′-UTR (e.g., ferritin and FPN1 mRNAs), IRP binding inhibits translation, thereby reducing iron storage and export. Conversely, when IREs are located in the 3′-UTR (e.g., transferrin receptor 1 and divalent metal transporter 1 mRNAs), IRP binding stabilizes the mRNA and increases protein synthesis, thereby enhancing iron uptake. Thus, under low intracellular iron conditions, cells increase iron uptake and reduce iron storage/export, whereas under high iron conditions, iron uptake decreases and iron storage increases.

Inside the body, the liver serves as the main storage site for the body’s excess iron [2,8]. As represented in Figure 2A, in response to iron levels, the liver produces hepcidin, a small peptide hormone that acts as the master regulator of systemic iron balance [2,8,13]. This elegant feedback loop ensures that systemic iron levels are maintained within a narrow physiological range. Hepcidin production in the liver is driven by two distinct iron signals: tissue iron levels and acute changes in circulating plasma iron [14]. Hepcidin functions by binding directly to the iron exporter FPN1, which is highly expressed on duodenal enterocytes and macrophages [8,13]. This binding event triggers the internalization and subsequent degradation of FPN1, effectively blocking the release of iron into the bloodstream [2,8,13]. Hepcidin is only regulated at the transcriptional level. Its expression is inhibited by anemia, hypoxia, and ineffective erythropoiesis, and stimulated by iron loading and inflammation [15]. When body iron stores are high, the liver increases hepcidin production, which reduces iron absorption and sequesters iron in macrophages. Hepcidin induction by inflammation seems to play a protective role against some infections by lowering available circulating iron for infecting agents. However, excessive hepcidin induction in chronic inflammatory diseases can contribute to iron-restricted erythropoiesis and anemia [14]. Conversely, iron deficiency and erythropoietic demand suppress hepcidin production by hepatocytes. In the absence of hepcidin, FPN1 is stabilized on the basolateral surface of duodenal enterocytes, iron-recycling macrophages, and hepatocytes. In these cells, it transports iron from the intracellular space to the plasma for loading onto Tf and delivery to red blood cells and other tissues [14].

### 2.3. The IRE/IRP System

Cellular iron homeostasis is tightly controlled by a post-transcriptional regulatory system that modulates the expression of key iron-related proteins according to intracellular iron availability (Figure 2B). This system relies on the interaction between iron regulatory proteins (IRPs) and iron-responsive elements (IREs), conserved RNA stem–loop structures located within the untranslated regions (UTRs) of target mRNAs. Under iron-deficient conditions, IRPs bind to IREs, whereas elevated cytosolic iron levels induce conformational changes in IRPs that prevent their association with RNA [16,17].

The functional outcome of IRP–IRE binding is determined by the position of the IRE within the transcript. In mRNAs encoding proteins involved in iron uptake, such as TfR and DMT1, IREs are located in the 3′-UTR, where IRP binding enhances mRNA stability and protects it from degradation. Conversely, transcripts encoding proteins involved in iron storage and export, including ferritin and FPN1, contain IREs in the 5′-UTR, where IRP binding inhibits translation initiation. Through this dual regulatory mechanism, the IRP–IRE network ensures coordinated control of iron acquisition, storage, and export in response to cellular iron status [16,17].

## 3. Cerebral Iron Metabolism

The entry of iron from the bloodstream into the unique microenvironment of the brain is a tightly regulated, multi-step transport system controlled by the blood–brain barrier (BBB). The principal pathway involves the binding of holo-Tf with TfR on the luminal (blood-facing) surface of brain microvascular endothelial cells and its internalization via endocytosis [5,8,18]. The iron is then trafficked to the abluminal (brain-facing) membrane and exported into the brain’s extracellular fluid by FPN1. There it is re-oxidized to Fe^3+^ by the copper-dependent ferroxidases ceruloplasmin and hephaestin [8]. Astrocyte end-feet, covering ~95% of brain capillaries, act as critical intermediaries in regulating cerebral iron uptake and metabolism at the BBB. The DMT1 and the TfR are intensely expressed in astrocyte end-feet contacting endothelial cells, suggesting that astrocytes can hypothetically absorb the Tf and non-Tf-bound iron (NTBI) directly from these cells [18,19]. In addition, astrocytes control iron entry into the parenchyma by secreting hepcidin, which regulates FPN1 on endothelial cells [5,18,20]. The choroid plexus acts as a sophisticated “gatekeeper” for the brain, utilizing a specific suite of proteins to manage iron levels. FTL exhibits prominent expression within the choroid plexus epithelial cells close to the vasculature. Conversely, FTH has been mainly observed in the outer membrane of the choroid plexus. In this location, FTH likely serves a neuroprotective role by shielding the brain parenchyma from oxidative insults caused by excess heme iron or other metals [21]. Moreover, both FPT and DMT1 proteins have been detected in the epithelial cells of the choroid plexus, where they can act as a regulatory checkpoint for iron transport between the brain and ventricles [21].

After entering the brain, iron binds to Tf, mainly secreted by epithelial cells of the choroid plexus [22]. Compared with the peripheral tissues, the concentration of NTBI in brain interstitial fluids is higher because citrate and ascorbate secreted by astrocytes help to maintain iron in the reduced Fe^2+^ status [23]. FPN1 is currently recognized as the only known mammalian protein capable of exporting ferrous iron from the intracellular environment to the extracellular space [24]. In the brain parenchyma, FPN1 cooperates with a membrane-anchored glycosylphosphatidylinositol-linked ceruloplasmin that, by converting Fe(II) into Fe (III), allows it to bind to Tf [25]. This coupled system is essential for maintaining iron homeostasis across various neural cell types. Both proteins are highly expressed on astrocytic membranes, where they facilitate the mobilization of iron into the extracellular space [24]. Ceruloplasmin has also been described to play an essential role in maintaining FPN1 stability at the cell surface, counteracting the effects of hepcidin, which instead promotes FPN1 internalization. Accordingly, mutated ceruloplasmin fails to properly stabilize FPN1, leading to its accelerated degradation, increased intracellular redox-active iron retention, and reduced iron availability to other cells [26]. Similar to its role in systemic iron balance, hepcidin serves as a central regulator of brain iron homeostasis, with its presence confirmed across diverse brain regions [27]. Current evidence indicates that the brain hepcidin pool is dual-sourced, arising from both in situ production by neurons and glia as well as from the systemic circulation [27]. While a compromised BBB markedly facilitates hepcidin translocation, mounting evidence suggests the peptide may also traverse a physiological, intact barrier, though this mechanism remains to be definitively established [27]. By interacting with FPN1 and promoting its degradation, hepcidin controls the export of iron from brain cells [27].

Once inside the brain, iron is distributed and handled by the different neural cell populations that constitute the functional architecture of the brain. Neurons and glial cells can autonomously modulate iron uptake, storage, and intracellular distribution in accordance with their specific functional demands, while also contributing to iron homeostasis across neighboring neural populations. As a result, precise regulation of iron acquisition, sequestration, trafficking, and release is required both at the level of individual cells and through coordinated interactions among distinct neural cell types.

### 3.1. Neurons

**Figure 3:**
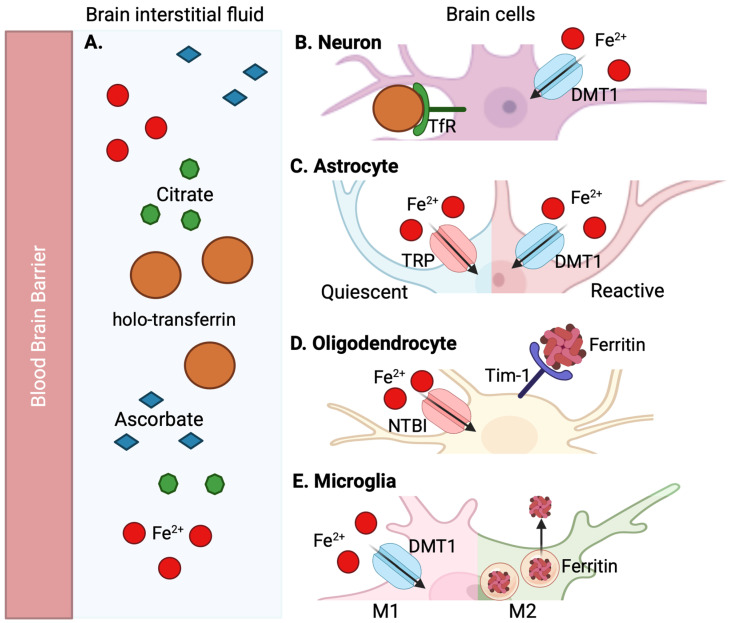
Iron uptake across different brain cells. (**A**) In the brain interstitial fluids, iron can bind to transferrin (Tf). However, the concentration of non-transferrin-bound iron (NTBI) is higher than in the peripheral tissues, because citrate and ascorbate secreted by astrocytes help to maintain iron in the reduced Fe^2+^ status. (**B**) Iron is taken up by neurons mainly through endocytosis of the transferrin/transferrin-receptor (Tf/TfR) complex, although Fe^2+^ can cross the plasma membrane via divalent metal transporter 1 (DMT1). (**C**) Astrocytes rely on NTBI as their main iron source. In their quiescent state, astrocytes handle iron via resident Transient Receptor Potential (TRP) channels. In contrast, in reactive astrocytes, newly expressed DMT1 becomes the dominant pathway for iron uptake. (**D**) Similar to astrocytes, mature oligodendrocytes do not express TfR and acquire iron primarily as NTBI from the extracellular fluid. They also express the T-cell immunoglobulin and mucin domain (Tim-1) receptor, which specifically binds and internalizes H-ferritin. (**E**) Microglial iron metabolism is linked to their activation state. In the anti-inflammatory state (M2), microglia can release iron, often packaged in ferritin, to support processes such as neuronal remyelination and repair following injury. In contrast, in a proinflammatory state (M1), microglia upregulate iron uptake machinery such as DMT1, leading to intracellular iron accumulation.

Neurons exhibit exceptionally high metabolic demands that necessitate a constant and reliable supply of iron for processes such as energy production and neurotransmitter synthesis. Neurons express high levels of TfR, establishing Tf-bound iron as their principal source, though they are also capable of taking up NTBI [8] (Figure 3B). Critically, this high-demand, multi-pathway influx system is paired with a limited capacity for safe intracellular storage since neurons contain the least amount of cytosolic ferritin, when compared to glial cells [28]. The paradox of high activity-dependent iron influx and low storage capacity is the core factor underpinning neuronal susceptibility to iron dyshomeostasis, necessitating robust buffering systems provided by surrounding glial cells.

### 3.2. Glial Cells

Glial cells are not passive bystanders in brain metabolism but are active and essential regulators of the brain’s iron landscape. Astrocytes, oligodendrocytes, and microglia each possess unique and complementary mechanisms to acquire, store, and distribute iron. Collectively, they work to support neuronal function and protect the entire central nervous system (CNS) from the potentially devastating effects of iron-induced damage.

#### 3.2.1. Astrocytes

Astrocytes play a pivotal role in maintaining the delicate balance of the brain’s microenvironment, acting as the primary regulators of the NTBI pool by releasing into the brain interstitial fluids ascorbate, citrate, and other carriers [29]. Unlike neurons or oligodendrocytes, astrocytes do not have an elevated metabolic iron requirement [19]. In the normal CNS, a limited number of astrocytes in discrete brain regions have been described to contain iron stored in ferritin, supporting the concept that astrocytes may be predominantly implicated in brain iron trafficking rather than iron storage [19]. Despite this observation, it has been shown that FTH expression in astrocytes is essential for oligodendrocyte iron accumulation and myelination during early development [19].

Unlike neurons, astrocytes do not express, in vivo, TfR and consequently rely on NTBI as their main iron source [29]. Their iron handling machinery is remarkably plastic, adapting its mechanisms based on the physiological state of the surrounding tissue (Figure 3C). In their quiescent state, astrocytes handle iron via resident Transient Receptor Potential (TRP) channels, which mediate the influx of NTBI, primarily in its ferrous (Fe^2+^) form [29]. Notably, the canonical iron transporter DMT1 does not play a significant role in this basal uptake process [29]. In contrast, during neuroinflammation, activated astrocytes shift their strategy dramatically in response to inflammatory cytokines such as TNF-α and IL-1β [29]. This activation triggers a distinct, inducible mechanism characterized by the de novo expression and massive upregulation of DMT1 [29]. In activated astrocytes, this newly expressed DMT1 becomes the dominant pathway for iron uptake, as confirmed by experiments showing that DMT1 blockers completely abolish the increased iron uptake, while TRP channel blockers have no effect [29]. This adaptive switch to a high-capacity, DMT1-mediated system represents a powerful neuroprotective emergency response. It allows activated astrocytes to function as highly efficient sinks, sequestering excess extracellular iron, thereby actively shielding vulnerable neurons from a potentially toxic iron overload [29].

#### 3.2.2. Oligodendrocytes

Oligodendrocytes have the highest iron concentration among all glial and neuronal cells in the brain, with levels roughly fivefold higher than neurons, threefold higher than microglia, and twofold higher than astrocytes [24,28]. Most of this iron is sequestered within ferritin cages, mainly composed of FTH, to prevent oxidative damage [28,30]. This high iron content is crucial for myelin synthesis and energy metabolism. Similar to astrocytes, mature oligodendrocytes do not express TfR and acquire iron primarily as NTBI from the extracellular fluid [31] (Figure 3D). However, they also possess a specialized acquisition pathway that is unique among the CNS cell types discussed. Oligodendrocytes express the T-cell immunoglobulin and mucin domain (Tim-1) receptor, which specifically binds and internalizes H-ferritin, allowing them to uptake iron already packaged within its storage protein [31]. This profound dependence on iron makes oligodendrocytes highly susceptible to damage from both iron deficiency, which leads to impaired myelination, and iron overload, which causes severe oxidative stress. In fact, oligodendrocytes have been described to have the highest level of oxidative metabolism of any cell per volume in the brain [16,31].

#### 3.2.3. Microglia

Microglia, the resident macrophages of the CNS, exert an integral role in brain iron homeostasis. They are equipped with a comprehensive suite of iron-handling proteins, including DMT1, TfR, ferritin in its FTL isoform, FPN1 for export, and the regulatory hormone hepcidin [13,21]. Fundamentally, microglial iron metabolism is linked to their activation state, or polarization, which shifts in response to the brain’s inflammatory status [13] (Figure 3E). Although the use of the terms “M1 versus M2” to indicate active or resting microglia oversimplifies a complex process for microglial activity [32,33], for the sake of simplicity, we will keep here this nomenclature. In response to injury or pathogens, microglia adopt a proinflammatory (M1-like) phenotype, which functions as an “iron sequestration” mode, limiting extracellular iron availability. M1 state is characterized by a hypertrophic morphology with shortened cellular branches, and it is induced via the activation of toll-like receptor (TLR) and NOD-like receptor pathways [34,35]. Activated microglia produce pro-inflammatory mediators, which contribute to the elimination of pathogens. M1 microglia secrete cytokines like the tumor necrosis factor alpha (TNF-α), interleukin-6 (IL-6), and interleukin-1 beta (IL-1β). Redox molecules are also released, including nitric oxide (NO), which is produced in large amounts from L-arginine by inducible nitric oxide synthase (iNOS) [34,35]. A key consequence of IL-6 release is the induction of hepcidin production. This effect occurs mainly indirectly by enhancing the activation of the Janus kinase signal transducers and activators of transcription (JAK/STAT) pathway [15]. Moreover, pro-inflammatory factors such as TNF-α and IL-1β induce microglia to upregulate iron uptake machinery like DMT1 while downregulating the exporter FPN1, leading to the accumulation of intracellular iron [36,37]. M1 microglia also secrete lactoferrin, an iron-binding protein with iron affinity about 300 times higher than Tf [37]. Altogether, these strategies are designed to limit iron availability to pathogens and fuel inflammatory processes. Conversely, during tissue repair and the resolution of inflammation, microglia shift towards an anti-inflammatory (M2-like) phenotype, which functions as an “iron donation” mode [36]. M2 microglia secrete mainly interleukin-4 (IL-4), interleukin-13 (IL-13), interleukin-10 (IL-10), and transforming growth factor beta (TNF-β), which antagonize the inflammatory response and reduce NO release [34,35]. While many pro-inflammatory responses act to promote iron sequestering, M2 cytokines inhibit pro-inflammatory cytokine receptors, thus reducing hepcidin expression and promoting iron secretion [15]. The iron release, often packaged in ferritin, is functional to support processes like neuronal remyelination and repair following injury [13,36]. This dual role highlights the delicate balance of microglial function. While they are more efficient at storing iron than other brain cells and can protect neurons from acute toxicity, the excessive accumulation of iron within microglia can trigger the chronic release of proinflammatory cytokines and reactive oxygen species (ROS), thereby contributing directly to neurodegeneration.

## 4. Physiological Functions of Iron

The sophisticated regulatory networks that maintain systemic and brain iron homeostasis exist precisely to safeguard the element’s vital role in cellular physiology. Iron’s essentiality is rooted in its remarkable electrochemical versatility, which allows it to serve as a critical cofactor in a wide range of proteins and enzymes that drive fundamental biological processes, as summarized in Table 1. Without a steady supply of iron, the most basic functions required for cellular life would be compromised.

### 4.1. Cellular Respiration and ATP Synthesis

Iron plays a central role in cellular respiration, the process by which cells convert nutrients into adenosine triphosphate (ATP). For this reason, tissues with high metabolic activity and oxygen consumption, such as the brain, are profoundly dependent on a consistent iron supply. Mitochondria are the primary intracellular sites of iron utilization. In the mitochondrial matrix, iron is required for the biosynthesis of heme and iron–sulfur (Fe–S) clusters, which act as cofactors for proteins involved in essential cellular processes such as electron transfer, energy production, and gene expression [38]. Fe–S clusters are composed of ferrous and ferric iron coordinated with inorganic sulfide, enabling electron delocalization across iron and sulfur atoms and conferring functional versatility [38]. Mitochondrial iron uptake relies on mitoferrin. While porins in the outer mitochondrial membrane (OMM) allow nonspecific diffusion of small molecules and ions, the inner mitochondrial membrane (IMM) is largely impermeable and requires specific transporters to deliver molecules to the matrix [38]. The presence of MtFt in the mitochondrial matrix highlights mitochondria as important sites of iron storage [38].

### 4.2. DNA Synthesis, Replication, and Repair

Iron is an essential cofactor for genomic stability, functioning as a critical component of the enzymatic machinery governing both DNA synthesis and repair. Its primary role in DNA synthesis is mediated through ribonucleotide reductase (RNR), which requires a di-iron center to generate the tyrosyl radical necessary for the rate-limiting conversion of ribonucleotides to deoxyribonucleotides (dNTPs). Beyond de novo nucleotide synthesis, iron in the form of (Fe-S) clusters is indispensable for the structural integrity and catalytic activity of key replication and repair enzymes. In these proteins, the Fe-S domains facilitate DNA binding and charge transport, linking cellular iron homeostasis directly to the maintenance of genome integrity [39].

## 5. Iron’s Specialized Roles in the Central Nervous System

The brain’s exceptionally high metabolic rate and oxygen consumption, accounting for approximately 20% of the body’s total energy use [40], create a profound and continuous dependency on iron. This high rate of metabolism is remarkably constant despite widely varying mental and motoric activity [40].

As indicated in Table 1, besides its role in energy production and genome stability, within the brain, iron also plays a pivotal role in specialized cerebral functions.

### 5.1. Biosynthesis of Neurotransmitters

Efficient neuronal communication, which underpins all brain functions, relies heavily on adequate iron availability. Iron acts as an essential cofactor for multiple enzymes involved in neurotransmitter biosynthesis and metabolism, including tyrosine hydroxylase, monoamine oxidase, and tryptophan hydroxylase [18]. In addition, iron deficiency has been linked to markedly reduced activity of glutamate dehydrogenase and GABA transaminase, enzymes that play central roles in the synthesis and turnover of the neurotransmitter GABA [36]. As a result, insufficient iron levels can disrupt neurotransmitter homeostasis, compromise neuronal communication, and ultimately contribute to increased vulnerability to neurological and neuropsychiatric disorders.

### 5.2. Myelin Formation and Maintenance

Oligodendrocyte progenitor cells, as well as mature myelinating oligodendrocytes, are the highest iron-rich cells in the brain [28]. Iron plays a direct and indispensable role in myelin formation. During embryonic brain development, the peak in iron uptake coincides with the initiation of myelination, suggesting that iron accumulation within oligodendrocytes is a critical requirement for proper myelin production [41]. Iron functions as an essential cofactor for several enzymes involved in lipid and cholesterol biosynthesis, including lanosterol 14α-demethylase, a cytochrome P450 enzyme that catalyzes key steps in cholesterol synthesis [41]. Because cholesterol is a fundamental structural component of myelin, its availability is vital for myelin membrane expansion. Additional iron-dependent or iron-associated enzymes are necessary for myelin synthesis and remodeling. These include squalene monooxygenase, which participates in cholesterol biosynthesis, lipid saturases and desaturases such as acyl-CoA desaturase, and lipid dehydrogenases, involved in lipid turnover [41]. Iron is also pivotal to the remyelination process, which necessitates the recruitment of oligodendrocyte progenitor cells to demyelinated lesions and their subsequent maturation into myelin-forming oligodendrocytes. Beyond its role in myelin formation, iron regulates a broad spectrum of functions within the oligodendrocyte lineage, including metabolism, proliferation, and differentiation, facilitating the deposition of new myelin membranes into lesions [41].

## 6. Role of Iron in Neurogenesis and Neurodevelopment

The essentiality of iron in general brain physiology underscores its even more profound involvement in the orchestrated processes of neurogenesis and neurodevelopment. Neurogenesis and neurodevelopment are highly coordinated processes that begin during embryogenesis and extend into the early postnatal period. While the majority of neuronal production is completed before birth, critical developmental events such as synaptogenesis, synaptic pruning, gliogenesis, and myelination continue postnatally [1,42]. Moreover, neurogenesis persists throughout adulthood in discrete brain regions, notably the subventricular zone (SVZ) lining the lateral ventricles and the subgranular zone (SGZ) of the dentate gyrus in the hippocampus [1,42]. These processes require the tightly regulated availability of iron to ensure proper brain structure and function. Iron demand is particularly high during periods of rapid brain growth, coinciding with intense neural stem cell proliferation, neuronal differentiation, synapse formation, and myelination [1]. At the cellular level, as noted above, iron is an essential cofactor for ribonucleotide reductase, thereby directly linking iron availability to DNA replication and cell-cycle progression in neural progenitor cells. Beyond its role in proliferation, iron critically influences neuronal lineage differentiation. For instance, an elevated iron concentration has been demonstrated to markedly accelerate the differentiation of human embryonic stem cells toward motor neuron lineage, potentially via a Tf-mediated pathway [43]. However, iron chelation with deferoxamine has also been shown to increase the differentiation rate of neuronal progenitor cells by inhibiting the Wnt/β-catenin pathway [44]. Along the same lines, iron accumulation in the hippocampus is reported to remarkably impair the differentiation of neural stem cells, resulting in a significant decrease in newborn neurons [45]. The damage was attributed to iron-induced downregulation of the proprotein convertase furin and subsequent decreased maturation of brain-derived neurotrophic factor, thus contributing to memory decline and anxiety-like behavior in mice [45].

Disruption of iron transport and regulation has profound consequences for neurodevelopment. For instance, the conditional deletion of the iron transporter Slc22a17 in the murine brain leads to early postnatal lethality, growth retardation, excessive neural stem cell apoptosis, and cognitive impairment [42]. Other hepcidin knockout mouse models exhibit impaired hippocampal neurogenesis and subsequent cognitive dysfunction [20,46]. Both phenotypes were associated with increased iron content after the depletion of hepcidin, followed by an elevated level of inflammatory TNF-a [46]. Overall, these findings emphasize that both iron deficiency and excess can have detrimental effects on neurogenesis.

## 7. Iron Deficiency and Neurodevelopmental Deficits

Building on the critical role of iron in healthy neurodevelopment, any disruption to its tightly regulated balance can have severe consequences. Specifically, iron deficiency (ID) during fetal, neonatal, and early postnatal life leads to neurodevelopmental deficits by disrupting the maturation of neural circuits underlying motor function, cognition, emotion, and behavior during critical periods of brain development. Because iron availability varies across developmental windows, the neurodevelopmental consequences of ID depend on which iron-dependent neural systems are undergoing rapid maturation at the time of deficiency [47]. The neurological consequences of early-life ID can be categorized into acute deficits, earlier postnatal ID effects, and long-term neurobehavioral alterations, reflecting the dependence of later-developing brain systems on earlier iron-dependent neural organization [47,48]. Natal ID acutely compromises recognition memory, neural processing speed, and neurological reflex integrity [3,47,48]. Motor dysfunction in particular is one of the earliest manifestations of ID-related neurodevelopmental impairment, reflecting disrupted maturation of motor pathways and sensorimotor integration [3]. Prenatal ID and anemia are associated with reduced gross and fine motor performance in offspring, likely due to impaired myelination, altered neurotransmission, and abnormal development of corticospinal and cerebellar circuits [3]. The severity of motor deficits depends on the timing and duration of ID. In particular, third-trimester deficiency is particularly detrimental, as it coincides with peak periods of myelination and motor system refinement [49]. ID also impairs learning and memory, most probably by disrupting hippocampal development and fronto-hippocampal connectivity, as revealed in animal models of ID [50,51]. These early deficits predict long-term impairments in attention, executive function, and planning during childhood and adolescence, demonstrating developmental cascading effects from early iron-dependent neural disruptions to later higher-order cognitive systems [47]. Postnatal ID further exacerbates these outcomes by inducing persistent deficits in cognition, socioemotional behavior, sleep regulation, and inhibitory control, most likely through sustained alterations in monoamine neurotransmission [48]. Fetal ID significantly elevates the risk of long-term postnatal psychopathologies, including autism, schizophrenia, and neurocognitive disorders [52,53]. The specific nature of these risks correlates with the timing of the maternal ID, depending on whether it occurred during the preconceptional period or within a specific gestational trimester [47].

Collectively, these findings indicate that ID impairs neurodevelopment by altering the formation, connectivity, and function of neural systems during critical periods. Consequently, this disruption leads to long-lasting motor, cognitive, and affective deficits that often persist despite later iron repletion.

From a mechanistic viewpoint, ID disrupts neurodevelopment through convergent effects on synaptic plasticity, neurotransmitter metabolism, myelination, cellular energy production, and gene regulation. The developing brain is a highly metabolic organ and, therefore, has exceptionally high iron requirements to support iron-dependent processes such as oxidative phosphorylation, neurotransmitter synthesis, and lipid metabolism. In addition to impairing mitochondrial energy production, ID can also affect epigenetic regulation of gene expression [47]. One major mechanism by which ID impairs neurodevelopment is through disruption of synaptic plasticity, the capacity of synapses to modify their structure and function in response to activity. Experimental models demonstrate that fetal ID induces persistent, region-specific alterations in dendritic morphology within the hippocampus and cortex, including reduced basal dendrite length and branching complexity, even after postnatal iron repletion [54,55]. These structural abnormalities are accompanied by reduced long-term potentiation and decreased expression of synaptic proteins critical for plasticity and vesicle release [56,57]. ID also impairs neurodevelopment by disrupting monoamine neurotransmitter systems, as iron is an essential cofactor for tyrosine and tryptophan hydroxylases required for dopamine and serotonin synthesis [18]. Moreover, in the striatum of rat brains, ID has been shown to reduce dopamine transporter density and receptor function, leading to impaired dopaminergic signaling [58]. As described above, these changes are associated with deficits in learning, memory, affect regulation, and increased risk of neuropsychiatric disorders. Myelination represents another iron-sensitive process underlying ID-related neurodevelopmental deficits, as iron-dependent desaturases are required for fatty acid synthesis and myelin lipid composition [41]. Oligodendrocytes have high metabolic and iron requirements, and ID reduces myelin basic protein and proteolipid protein expression, resulting in long-lasting hypomyelination and reduced neural conduction speed [41]. These alterations likely contribute to the slower processing speed observed in iron-deficient neonates, infants, and toddlers.

Overall, these mechanisms demonstrate that ID during fetal and early postnatal life impairs neurodevelopment by targeting multiple interdependent biological systems during sensitive windows of brain maturation, leading to long-term and often irreversible functional consequences.

## 8. Iron Accumulation and Neurodegenerative Disorders

While iron is essential for neurodevelopment and its deficiency is associated with neurodevelopmental deficits, dysregulated accumulation of iron can be toxic as well, contributing to ROS generation. Although ROS at low levels play physiological functions, modulating, for instance, neural differentiation and maturation, excessive levels cause neuronal damage and are associated with neurodegenerative disorders, including Alzheimer’s disease (AD), Parkinson’s disease (PD), Neurodegeneration with Brain Iron Accumulation (NBIA), and Amyotrophic Lateral Sclerosis (ALS). While iron is unlikely to be the initial trigger of neurological disorders, its accumulation may accelerate disease progression. This is supported by the presence of the iron-storage protein FTL in both AD senile plaques and PD Lewy bodies [21].

### 8.1. Iron Dyshomeostasis in Alzheimer’s Disease

AD is a progressive neurodegenerative disorder clinically characterized by memory impairment and cognitive decline. Pathologically, AD is defined by extracellular β-amyloid (Aβ) plaques, intracellular neurofibrillary tangles composed of hyperphosphorylated tau, and widespread neuronal loss [16,59]. Although the precise etiology of AD remains unresolved, substantial evidence indicates that brain iron accumulation and dysregulation of iron metabolism, accompanied by increased ROS generation, play a central role in disease pathogenesis [16,59]. Iron deposition within Aβ plaques and neurofibrillary tangles has been consistently reported in postmortem AD brains, indicating disrupted iron homeostasis in affected regions [60]. Early imaging studies further demonstrated that iron accumulation colocalizes with Aβ aggregation in the cerebral cortex and hippocampus during the initial stages of AD, preceding overt neurodegeneration [61]. Compared with healthy individuals, AD patients exhibit significantly elevated iron levels in memory-related brain regions, where excess iron promotes Aβ aggregation, enhances oxidative stress, and accelerates neuronal death [61].

Iron levels also influence amyloid precursor protein (APP) expression and processing, which critically determines Aβ generation. Under physiological conditions, APP is predominantly cleaved by α-secretase and γ-secretase, whereas increased iron downregulates α-secretase activity and shifts APP processing toward the β-secretase pathway that generates Aβ [16,59]. At the translational level, APP expression is regulated by an IRE in its 5′-UTR, whereby high intracellular iron disrupts IRP binding and increases APP synthesis, further enhancing Aβ production [59]. Interestingly, APP also plays an important role in neuronal iron export by interacting with FPN1 and exhibiting ferroxidase activity required for safe iron efflux and Tf loading [16]. Altered APP processing in AD may therefore impair iron export, leading to intracellular iron retention and oxidative stress.

Iron directly induces Aβ aggregation and amplifies its neurotoxicity by catalyzing ROS production, thereby reinforcing a feed-forward cycle of oxidative damage and protein misfolding in AD brains [16,59]. As previously mentioned, Aβ and the iron storage protein ferritin have been shown to co-localise in the vascular amyloid deposits in post-mortem AD brains [21]. Extensive blood vessel damage and a reduction in hepcidin and FPN1 levels in the AD brain have also been reported [62]. Interestingly, in a mouse model, astrocyte-overexpressed hepcidin has been demonstrated to decrease brain iron levels, possibly by acting on FPN1 on the brain microvascular endothelial, and to protect against Aβ-induced cortical and hippocampal damages [63].

### 8.2. Iron Dyshomeostasis in Parkinson’s Disease

PD is a common neurodegenerative disorder pathologically defined by the progressive loss of dopaminergic neurons in the substantia nigra pars compacta (SNpc), and the presence of Lewy bodies composed mainly of aggregated α-synuclein [64]. A consistent pathological feature of PD is the abnormal accumulation of iron in the SNpc, which has been documented in postmortem brains and experimental models [64]. The SNpc is physiologically enriched in iron, most probably due to the high metabolic activity of dopaminergic neurons, their requirement for iron in dopamine synthesis, and the presence of neuromelanin, which binds iron and normally limits its redox activity [64]. Disruption of iron homeostasis in this vulnerable region can therefore have disproportionate toxic effects, promoting oxidative stress, mitochondrial dysfunction, and neuronal death. Dopamine metabolism further amplifies iron toxicity, as dopamine auto-oxidation and monoamine oxidase activity generate reactive hydrogen peroxide, which reacts with iron via Fenton chemistry to produce highly damaging hydroxyl radicals [64]. Although neuromelanin normally sequesters redox-active iron and exerts neuroprotective effects, its release in the extracellular space from degenerating neurons activates microglia. This process fuels chronic neuroinflammation and further links iron dysregulation to progressive neurodegeneration [64].

Iron can also directly contribute to α-synuclein pathology, as both iron and α-synuclein accumulate within Lewy bodies, and elevated iron levels accelerate α-synuclein aggregation [65]. As in the case of APP, regulation of α-synuclein expression is partly mediated by an IRE in its 5′-UTR, implicating iron-regulatory protein signaling in post-transcriptional control [66].

By comparing the distribution and expression of key iron proteins, levels of both FTL and FTH in the basal ganglia were shown to be strongly reduced in PD brains than in control ones [21]. Moreover, similarly to AD, lower levels of FPN1 and hepcidin, together with damaged blood vessels, were also observed in PD brains compared to controls [21]. Notably, disruption of the BBB and microvascular pathology are considered important contributors to neurodegenerative disorders [67]. Accordingly, in control brains, DMT1 expression was observed in the vascular endothelium, accompanied by glial fibrillary acidic protein (GFAP)-positive astrocytic processes. These structures likely facilitate protein uptake and transport into the brain parenchyma. In contrast, PD brains were described as exhibiting marked vascular damage, with significant loss of endothelial structural integrity [21].

### 8.3. Iron Dyshomeostasis in Neurodegeneration with Brain Iron Accumulation

NBIA comprises a group of rare inherited neurodegenerative disorders defined by pathological iron deposition in specific brain regions, particularly the globus pallidus and substantia nigra [38]. NBIA affects approximately two individuals per million and presents with marked clinical heterogeneity, typically with early childhood onset and rapid progression, although atypical late-onset and slowly progressive forms have also been described [38]. Clinically, NBIA is characterized by movement disorders such as dystonia and dysarthria. It may then progress to spasticity, chorea, and parkinsonism, often leading to loss of ambulation within 15 years. Additional oculomotor, retinal, cognitive, and gastrointestinal manifestations also occur, and they contribute significantly to morbidity and mortality [38]. At the neuropathological level, iron accumulation in NBIA is observed in neurons, microglia, macrophages, and extracellular spaces surrounding blood vessels, with the globus pallidus consistently showing the highest iron burden across all subtypes [38]. The substantia nigra pars reticulata is also frequently affected. Neuroferritinopathy and aceruloplasminemia are the only NBIA subtypes in which a more widespread iron deposition occurs. This deposition involves the liver and additional brain regions, including the striatum, dentate nuclei, thalamus, and red nuclei [38]. Importantly, these NBIA forms are the only ones linked to proteins directly involved in iron homeostasis: ceruloplasmin and FTL.

Mutations in the ceruloplasmin gene result in loss of ferroxidase activity, leading to defective iron export. This causes pronounced iron accumulation in the basal ganglia, thalamus, and cerebellum. It also drives downstream oxidative stress and lipid peroxidation, as shown in patient tissues, cell models, and knockout mice [68,69]. Mutations in the FTL gene affect ferritin structure, inducing ferritin aggregation and disrupting iron storage capacity. Similarly to aceruloplasminemia, this promotes iron overload, reactive oxygen species generation, and lipid peroxidation, as demonstrated in patient-derived cells and animal models [70].

Although iron accumulation is a defining diagnostic feature of NBIA, most genes associated with NBIA are not directly linked to iron metabolism. This leaves open the question of whether iron overload acts as a primary driver of the disease or arises as a secondary consequence of broader cellular dysfunction. As highlighted in a recent review [38], studies examining the cellular mechanisms underlying different NBIA subtypes indicate that iron deposition is more likely a characteristic hallmark rather than a shared causative factor of the pathological phenotype. Disruptions in lipid metabolism, as well as mitochondrial and lysosomal impairments, may instead represent key initiating events in disease onset and progression, with subsequent iron accumulation. Nonetheless, because these processes are highly interconnected, additional research is needed to fully clarify their relationships [38].

### 8.4. Iron Dyshomeostasis in Amyotrophic Lateral Sclerosis

ALS is a neurodegenerative disorder marked by the selective loss of motor neurons in the motor cortex, brainstem, and spinal cord [71]. Neuroimaging and neuropathological investigations have demonstrated pronounced iron accumulation in vulnerable regions in both ALS patients and experimental models, supporting a potential contribution of ferroptosis to the characteristic pattern of motor neuron degeneration observed in the disease. Magnetic resonance imaging studies have identified abnormal hypointense signals in the primary motor cortex of ALS patients, which histopathological analyses have linked to iron-enriched microglia located in the deep cortical layers [72,73,74]. Notably, iron accumulation in the motor cortex was found to correlate with upper motor neuron dysfunction and was detectable even at early, premanifest stages of ALS [72]. In addition, elevated levels of ferritin, a protein involved in iron storage, were described in the cerebrospinal fluid and serum of ALS patients, and they correlate with disease progression [75,76]. In transgenic ALS mouse models harboring SOD1 mutations, ventral horn motor neurons exhibit progressive iron accumulation that parallels disease advancement [77]. Collectively, these observations indicate increased iron loading in regions undergoing neurodegeneration, highlighting iron dysregulation as a potential contributor to ALS pathophysiology.

### 8.5. Iron in Friedreich’s Ataxia

Friedreich’s Ataxia is a neurodegenerative disorder primarily affecting the dorsal columns, nucleus dorsalis, corticospinal tracts, spinocerebellar tracts, dentate nuclei of the cerebellum, dorsal root ganglia, and sensory peripheral nerves. However, the pathology is not confined to the CNS, as cardiac and skeletal muscle involvement is also common. Accordingly, Friedreich’s Ataxia is currently recognized as a multisystem disorder [78]. The disease is caused by mutations in the frataxin gene, resulting in reduced expression of frataxin, a small mitochondrial protein that acts as an allosteric regulator of the iron-sulfur cluster biosynthetic machinery [79]. As previously mentioned, Fe–S clusters are major components of mitochondrial iron utilization, and it is therefore unsurprising that their dysfunction has profound effects not only on mitochondrial oxidative respiration but also on mitochondrial iron homeostasis. This notion is further supported by the ability of frataxin to bind both ferrous and ferric ions. Moreover, alterations in frataxin levels or activity affect the regulation of the IRP/IRE system, thereby disrupting overall mitochondrial iron metabolism [79,80].

Altered iron regulation has been reported in multiple models of Friedreich’s Ataxia, including frataxin-deficient flies, mice, and patients, where frataxin downregulation leads to dysregulated iron metabolism and mitochondrial iron accumulation [80]. Iron accumulation has been specifically observed within mitochondria, whereas cytosolic iron levels remain unchanged. This phenotype has been linked to impaired mitochondrial iron sensing caused by frataxin deficiency, which promotes increased expression of iron uptake proteins, such as TfR and DMT1, while reducing the expression or activity of iron-utilizing proteins, including ferritins and aconitase, through mechanisms that are still unclear [81]. Moreover, constitutive activation of the iron-deficiency response pathway has been shown to reduce the expression of key mitochondrial metabolic enzymes, including aconitase 2 and components of mitochondrial complex I [81].

Unlike the pathologies discussed previously, iron dyshomeostasis in Friedreich’s Ataxia appears to be one of the primary drivers of neurodegeneration, rather than merely a contributing factor or secondary consequence of disease progression. Furthermore, iron overload in this disease appears to be largely compartmentalized within mitochondria, underscoring the importance of tightly regulated iron homeostasis at the subcellular level. They further suggest that iron dyshomeostasis confined to a single organelle may be sufficient to compromise overall cellular viability.

### 8.6. Iron Dyshomeostasis in Wilson Disease

Wilson disease (WD) is a rare autosomal recessive disorder caused by mutations in the ATP7B gene, which encodes a copper-transporting ATPase. Impaired function of this protein leads to reduced biliary copper excretion and consequent pathological copper accumulation, primarily in the liver and the brain [82].

Clinically, WD most commonly presents with hepatic dysfunction. However, neurological manifestations represent a common feature of the disease. Neuropathological alterations predominantly affect the basal ganglia, particularly the putamen, and include a spectrum of tissue damage ranging from mild degeneration and to extensive necrosis. In the advanced stages, tissue cavities that may involve large portions of the putamen are visible in postmortem brain tissues. These degenerative changes are accompanied by pronounced astrocytic abnormalities, including reactive astrogliosis and the presence of enlarged and swollen astrocytes [83].

The neurodegenerative phenotype in WD is primarily attributed to copper-induced toxicity in extrahepatic tissues. Nevertheless, analyses of postmortem brain samples have also revealed alterations in iron homeostasis, characterized by both extracellular and intracellular iron accumulation, particularly in phagocytic cells and reactive astrocytes [83,84]. This observation is consistent with the well-established interplay between copper and iron metabolism; indeed, primary copper overload may promote secondary iron accumulation, a hypothesis supported by evidence showing that anti-copper therapies can partially reduce iron deposition in WD patients [85]. Interestingly, plasma analyses from WD patients presenting neurological symptoms revealed elevated levels of lipid peroxidation, a hallmark of ferroptosis, an iron-dependent form of regulated cell death that will be discussed in the following sections [86]. These findings further support the involvement of iron dysregulation in the neurological manifestations of the disease [86]. Overall, WD provides a clear example of how dysregulation of one metal can perturb the homeostasis of others. Secondary iron accumulation arising from primary copper dysregulation may exacerbate tissue damage and contribute to disease progression, highlighting the complex interdependence of metal metabolism in neurodegenerative disorders.

Altogether, the neurodegenerative diseases discussed here demonstrate that disruption of iron homeostasis is highly detrimental to neuronal health. Iron imbalance may arise either as a primary pathogenic event or as a secondary consequence of disease progression; nevertheless, in both contexts, it can significantly contribute to neuronal dysfunction and degeneration. Notably, iron accumulation has been observed in different neurodegenerative diseases characterized by distinct pathological features, affected brain regions, and clinical manifestations. This suggests that the consequences of iron dyshomeostasis may depend on the interplay between genetic factors, disease-specific molecular mechanisms, and the particular cell types or brain regions that are preferentially affected by iron overload. In this context, neuronal populations and glial cells may exhibit different susceptibilities to iron-mediated toxicity, potentially contributing to the heterogeneity observed among neurodegenerative diseases.

## 9. Mechanism of Iron Toxicity

While the clinical and pathological manifestations of iron dysregulation vary across these neurodegenerative disorders, the underlying driving force is rooted in the fundamental biochemistry of iron toxicity. The cellular processes involving the numerous iron-dependent enzymes rely on iron’s capacity to oscillate between its ferrous (Fe^2+^) and ferric (Fe^3+^) redox states via electron transfer [87]. Yet, the high reactivity of Fe^2+^, which renders iron indispensable for survival, also poses a potential threat to the cell. Alterations in iron homeostasis can result in an accumulation of free Fe^2+^, which interacts with hydrogen peroxide (H_2_O_2_) through the Fenton and Haber–Weiss pathways. The consequences of these chemical events are highly reactive hydroxyl radicals (●HO) that can damage proteins, nucleic acids, and membrane lipids, eventually causing cell death [88]. In addition to this increase in ROS production, which can foster a broad cellular effect, accumulation of iron is also involved in two more specific processes, namely ferroptosis and neuroinflammation, that can promote and fuel neurodegeneration.

### 9.1. Iron and Ferroptosis

**Figure 4:**
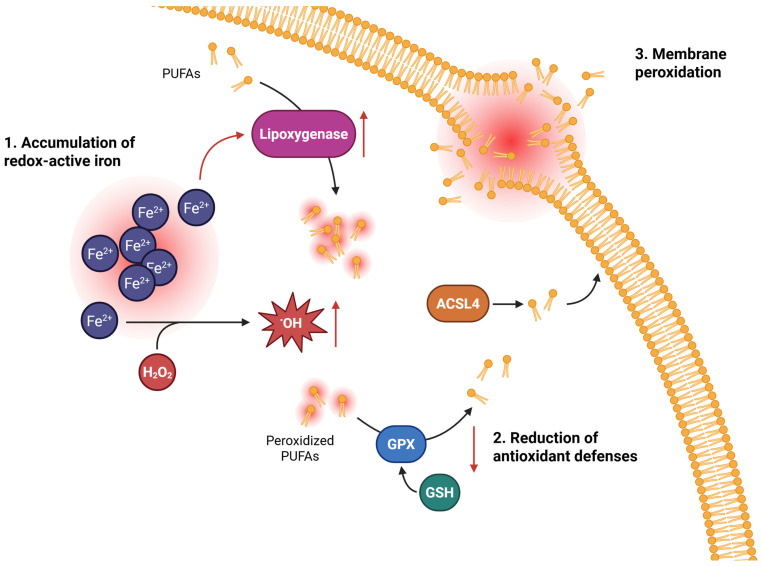
Schematic of key molecular mechanisms driving ferroptosis. (1) Ferrous iron fuels ferroptosis by reacting with hydrogen peroxide, generating hydroxyl radicals. Moreover, it stimulates lipoxygenases, iron-dependent enzymes that oxidize polyunsaturated fatty acids (PUFAs). This iron-driven chemistry amplifies reactive oxygen species (ROS) production, creating a self-propagating oxidative environment that rapidly overwhelms cellular redox balance. (2) A reduction in the antioxidant system composed of glutathione peroxidase 4 (GPX4) and glutathione (GSH) is another key driver of ferroptosis. GPX4 converts peroxidized PUFAs into non-toxic lipid alcohols and requires GSH as an essential reducing cofactor. Loss of GSH availability or direct inhibition/inactivation of GPX4 leads to unchecked accumulation of lipid hydroperoxides, removing the primary enzymatic brake on ferroptotic signaling. (3) Membrane lipid peroxidation is another hallmark of ferroptosis. In this context, PUFA-activating enzymes such as ACSL4 are particularly relevant, as they regulate the synthesis and incorporation of PUFA-containing phospholipids into cellular membranes. These PUFA-enriched membrane phospholipids serve as highly oxidation-prone substrates, making cellular membranes increasingly vulnerable to iron-dependent peroxidative damage and ultimately structural collapse.

In 2012, iron toxicity was linked to ferroptosis, a form of regulated cell death differing from apoptosis and other necrosis types [89]. Ferroptosis is defined by three primary features: (i) the existence of redox-active iron, (ii) the depletion of antioxidant defenses, specifically glutathione (GSH) and glutathione peroxidase 4 (GPX4), and (iii) the peroxidation of polyunsaturated fatty acid (PUFA)-rich membranes (Figure 4). Ferrous iron drives ferroptosis by fueling Fenton and Haber–Weiss chemistry and/or stimulating lipoxygenases, iron-dependent enzymes that oxygenate PUFAs like arachidonic acid [16,38]. Beyond sequestering free cytosolic iron in the cytosolic labile iron pool to halt oxidation, GSH serves as a cofactor for GPX4, converting PUFA-hydroperoxides into alcohols. Because membrane PUFA peroxidation is a hallmark of this process, enzymes handling PUFA membrane insertion are critical. Consequently, Acyl-CoA synthetase long-chain 4 (ACSL4) is recognized as a vital execution component [90]. ACSL4 drives PUFA-CoA formation, mainly regarding arachidonic acid, for esterification into PUFA-phospholipids [90]. Importantly, neurons in the brains of AD, PD, and ALS patients present multiple hallmarks of ferroptotic stress in affected regions, including increased lipid peroxidation products and disrupted expression of proteins that regulate iron homeostasis [91,92,93].

### 9.2. Iron and Neuroinflammation

**Figure 5:**
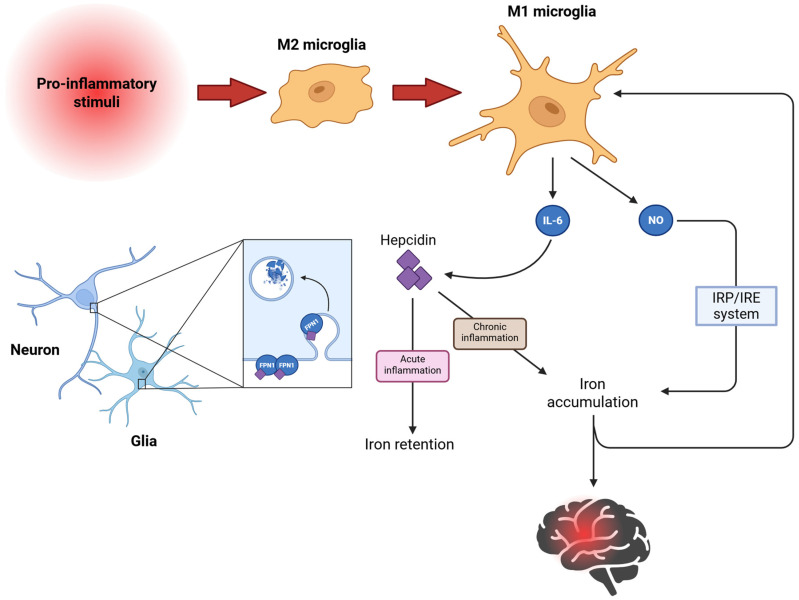
Mechanistic schematic of inflammation-driven iron accumulation in the brain. Pro-inflammatory stimuli promote microglial polarization toward the M1 phenotype, leading to the release of several pro-inflammatory cytokines, such as interleukin-6 (IL-6) and nitric oxide (NO). This M1-dominant state is characterized by sustained neuroinflammatory signaling and amplification of oxidative stress within the central nervous system. Among the secreted mediators, IL-6 induces hepcidin expression in the central nervous system, triggering ferroportin (FPN1) internalization and degradation, and consequently iron retention in neurons and glial cells. This hepcidin–FPN1 axis effectively blocks cellular iron export, causing progressive intracellular iron sequestration across multiple brain cell types. During chronic inflammation, the sustained hepcidin production and the persistent dysregulation in iron metabolism result in a pathological steady state in which iron progressively accumulates in the brain. In parallel, NO modulates the IRP/IRE pathway independently of iron-dependent feedback, further enhancing intracellular iron levels. This nitric oxide–mediated disruption of iron-sensing machinery decouples iron regulatory protein activity from physiological iron status, leading to inappropriate upregulation of iron import and reduced export. Increased iron accumulation promotes the shift from the anti-inflammatory M2 phenotype to the pro-inflammatory M1 phenotype, enhancing ROS generation and iron dyshomeostasis, thus reinforcing a self-perpetuating inflammatory cycle.

Neuroinflammation is defined as an inflammatory response within the CNS caused by various exogenous or endogenous factors. Microglia, the resident immune cells of the CNS, are central regulators of inflammatory signaling. While acute microglial activation is beneficial for debris clearance and tissue repair, chronic activation contributes to sustained neuroinflammation and neuronal damage in neurodegenerative diseases [94]. Strong evidence supports the involvement of chronic neuroinflammation in the pathogenesis of neurodegenerative disorders [95,96]. There is a bidirectional relationship between microglial iron accumulation and inflammation, creating a cycle that jointly promotes the progression of neurodegenerative disorders. Indeed, as outlined in Figure 5, neuroinflammation and iron dyshomeostasis are entangled in a circuit that amplifies ROS production, leading to neuronal death [97].

Pro-inflammatory stimuli promote microglial polarization toward an M1 phenotype characterized by the release of IL-1β, IL-6, TNF-α, ROS, and reactive nitrogen species (RNS) [94,98]. Among these mediators, IL-6 plays a pivotal role in linking inflammation to iron metabolism. Following microglia activation, IL-6 induces hepcidin expression in the CNS, particularly in astrocytes [98,99]. While increased hepcidin levels can initially exert neuroprotective effects by downregulating pro-inflammatory cytokines, thereby priming the brain’s cellular milieu against the deleterious effects of inflammatory iron overload, sustained hepcidin upregulation during chronic neuroinflammation triggers FPN1 internalization and degradation, limiting iron efflux and promoting intracellular iron retention in neurons and glial cells. This leads to pathological iron accumulation, oxidative stress, and neuronal vulnerability [98]. This delicate balance is further complicated because inflammatory mediators, especially NO, can target the IRP/IRE system and reshape iron homeostasis [98]. In fact, through the iNOS-dependent production of NO, microglia trigger the conversion of c-aconitase to IRP1 by disassembling its [4Fe-4S] cluster, leading to an overactivation of IRP1 even under iron-sufficient conditions [98]. This creates a positive feedback loop where inflammatory cytokines enhance iron accumulation, generating iron overload and subsequent cell death.

In turn, iron plays a pivotal role in modulating microglial differentiation between the pro-inflammatory M1 and the anti-inflammatory M2 phenotypes [98]. Iron overload triggers M1 polarization via a ROS-mediated mechanism, increasing the secretion of TNF-α and IL-1β, and causes M2 macrophages to switch their phenotype to M1 [100,101]. This iron-induced shift to an M1 phenotype has been suggested to represent an adaptive mechanism allowing microglia to survive in a pro-oxidant environment [98]. Accordingly, M2-polarized microglia appear more sensitive to ferroptosis because they possess increased levels of 15-lipoxygenase, an enzyme that catalyzes the production of essential pro-ferroptosis lipid signals [102,103]. However, the release of cytokines and ROS/RNS by activated microglial cells fuels the neuroinflammatory process and promotes neurodegeneration.

As aforementioned, a leaky BBB is susceptible to facilitating the entry of many macromolecules and cytokine/chemokines into the brain parenchyma, which can lead to neuroinflammation [21].

## 10. Imaging and Targeting Brain Iron

The accumulation of iron in specific brain regions observed in many neurodegenerative disorders can be used as a biomarker to detect disease progression. In this frame, advanced Magnetic Resonance Imaging (MRI) techniques function as non-invasive “biochemical sensors,” enabling the detection and precise quantification of pathological iron accumulation. MRI-based iron imaging has evolved from early T2-weighted hypointensity correlations to the utilization of R2* relaxation rates as a sensitive linear marker for tissue iron concentration. Currently, Susceptibility-Weighted Imaging (SWI) and Quantitative Susceptibility Mapping (QSM) represent the clinical standards for assessing cerebral susceptibility across diverse pathologies, including neurodegeneration and vascular disruption [104]. While SWI excels in providing qualitative contrast enhancement and structural delineation, QSM offers a superior, reproducible framework for the precise spatial quantification of brain iron distribution.

The recognition of iron as both a driver and a by-product of neuropathology opens new potential therapeutic avenues. In fact, the therapeutic targeting of brain iron dyshomeostasis has emerged as a significant strategy for mitigating neurodegeneration, primarily by suppressing ferroptosis and reducing oxidative stress. Recent research highlights the potential of iron chelators, such as deferiprone and deferoxamine, to reduce brain iron levels in PD and AD [105,106]. Innovative delivery methods, including intranasal administration, are currently being investigated to bypass the blood–brain barrier and minimize systemic side effects like neutropenia or peripheral iron depletion [107]. Table 2 summarizes the main clinical trials investigating the use of deferiprone for the treatment of PD and AD.

While initial observations were encouraging, showing reduced brain iron accumulation and some improvement in disease symptoms, significant limitations have emerged in recent large-scale clinical trials. Recent randomized controlled trials of deferiprone in patients with AD and PD have unexpectedly reported worsened clinical outcomes and accelerated cognitive decline despite a successful reduction in brain iron [110,111,112]. These findings suggest that high-affinity chelation may inadvertently disrupt essential iron-dependent physiological processes, such as mitochondrial function or neurotransmitter synthesis. They thus highlight the need for standardized regimens of chelator treatment, as well as a deeper understanding of iron’s role in neurodegeneration.

Importantly, QSM offers a non-invasive tool to guide therapeutic monitoring by quantifying iron burden before and after intervention, thereby enabling patient stratification and individualized treatment [104].

## 11. Conclusions

As highlighted throughout this review, iron redox capacity makes it essential for fundamental neurobiological processes, yet this same chemical reactivity can cause significant damage when homeostatic mechanisms are disrupted.

Iron deficiency during fetal and early postnatal life can disrupt the structural organization of the developing brain, leading to long-lasting impairments in motor function, cognition, and affective regulation. These early-life deficits highlight the importance of adequate iron availability to ensure the proper formation and refinement of neural circuits. Conversely, the aging brain and various pathological states reveal the adverse effects of iron dyshomeostasis. When the brain’s buffering capacity is exceeded or regulatory proteins fail, excess labile iron catalyzes the production of reactive oxygen species via Fenton and Haber–Weiss chemistry.

While iron accumulation is consistently associated with neurodegenerative disorders, a major unresolved question remains whether iron dyshomeostasis acts primarily as an initiating factor, a secondary consequence, or both, depending on disease stage and context. The recent clinical failures of broad-spectrum iron chelators like deferiprone demonstrate that removing cerebral iron is a blunt strategy that risks inducing localized iron starvation and disrupting vital physiological enzymes.

The growing recognition of ferroptosis and iron-driven neuroinflammation also opens promising avenues for future research. However, selective modulation of these pathways remains challenging due to the essential physiological functions of iron. Further work is needed to identify therapeutic targets capable of reducing toxic iron reactivity without impairing normal cellular metabolism. In this regard, the development of cell-specific delivery systems, targeted antioxidants, ferroptosis inhibitors, and modulators of microglial activation represents a particularly important direction. Moreover, advances in high-resolution imaging and omics technologies may improve the identification of early biomarkers of cerebral iron dysregulation, enabling earlier diagnosis and personalized therapeutic approaches.

Ultimately, future research should aim to integrate developmental neuroscience, neuroimmunology, and iron metabolism into a unified framework capable of explaining how iron contributes to both neural resilience and vulnerability across the lifespan. A deeper understanding of these interconnected mechanisms will be essential for designing interventions that preserve brain health while minimizing the detrimental consequences of iron imbalance.

## Figures and Tables

**Table 1:** Physiological functions of iron.

Function Category	Specific Role
Iron general physiological function	Heme and iron–sulfur (Fe–S) clusters in mitochondria, where it is required for electron transfer and energy production.
	Iron acts as cofactor of the enzymatic machinery governing DNA synthesis and repair.
Iron in the Central Nervous System	Cofactor for multiple enzymes involved in neurotransmitter biosynthesis and metabolism.
	Cofactor for enzymes important for lipid and cholesterol biosynthesis, essential myelin component.
	Regulations of Oligodendrocytes metabolism, proliferation, and differentiation, facilitating remyelination.
Iron in Neurogenesis and Neurodevelopment	Cofactor for ribonucleotide reductase, essential for DNA replication and cell-cycle progression in neural progenitor cells.
	Participation in neuronal lineage differentiation.

**Table 2:** Clinical trials using deferiprone to treat PD or AD.

Clinical Trial	Disease Treated	Key Findings in Deferiprone-Treated PD Patients Compared to Controls (Placebo)	Reference
NCT00943748	PD	Potential benefits: lower iron accumulation and slower disease progression	[108]
NCT01539837	PD	Short-term treatment is safe with reduced iron concentrations in specific brain regions.	[109]
NCT02655315	PD	Decreased nigrostriatal iron content. No benefits: some evidence of clinical worsening and some adverse events	[110]
NCT02728843	PD	No improvements in disease symptoms	[111]
ACTRN12617001578392	PD	Disease worsening	[111]
NCT03234686	AD	Decreased brain iron accumulation, but accelerated cognitive deterioration	[112]

## Data Availability

No new data were created or analyzed in this study.
